# Provision of injectable contraceptives by community health workers in sub-Saharan Africa: a systematic review of safety, acceptability and effectiveness

**DOI:** 10.1186/s12960-022-00763-8

**Published:** 2022-09-05

**Authors:** Besong Eric Ayuk, Brenda Mbouamba Yankam, Farrukh Ishaque Saah, Luchuo Engelbert Bain

**Affiliations:** 1 Human Resource Department, Ministry of Public Health, Yaoundé, Cameroon; 2 University of Nigeria, Nsukka, Nigeria; 3 Cameroon Baptist Convention Health Services, Yaounde, Cameroon; 4 School of Public Health, University of Health and Allied Sciences, Hohoe, Ghana; 5 Lincoln International Institute for Rural Health, College of Social Science, University of Lincoln, Lincolnshire, UK

**Keywords:** Community health workers, Injectable contraceptives, Depot-medroxyprogesterone acetate, Sub-Saharan Africa

## Abstract

**Background:**

Injectable contraceptives are the most popular method of contraception in sub-Saharan Africa (SSA), but their availability in clinical settings has been severely limited, despite the scarcity of health care providers and limited access to health facilities. WHO and USAID have endorsed the community-based distribution of injectable contraceptives as a promising option for improving access to family planning services and expanding the method mix for women who want to limit the number of births. Studies have shown that community health workers (CHWs) can provide women with injectable contraceptives that meet acceptable quality standards. The goal of this study is to identify, evaluate and synthesize evidence supporting the use of community-based administration of injectable contraceptives in SSA.

**Methods:**

This review's guidance was based on a previously developed protocol. Nine international electronic databases and the websites of organizations known to support community-based reproductive health initiatives in SSA were searched systemically. Experts in this area were also contacted for the identification of unpublished literature and ongoing studies. The reference lists of eligible studies were reviewed. The Effective Public Practice Project tool was used to assess the quality and risk of bias in eligible studies. Data were extracted and analysed using a custom data extraction form and a narrative synthesis.

**Results:**

The search strategy identified a total of 1358 studies with 12 studies meeting the inclusion criteria. One unpublished study was provided by an expert making a total of 13 studies. The results showed that irrespective of the study designs, well-trained CHWs can competently administer injectable contraceptives safely and community-based delivery of injectable contraceptives is acceptable in SSA. Also, the use of community health workers in the provision of depot-medroxyprogesterone acetate expanded access to inhabitants of hard-to-reach areas and led to an overall uptake of injectable contraceptives as well as family planning. Studies that compared CHWs to clinic-based providers revealed equivalent or higher levels of performance in favour of CHWs.

**Conclusions:**

The CHWs can competently provide injectable contraceptives within SSA communities if appropriately trained and supervised. Hence, SSA policymakers should give this initiative due consideration as a way of improving access to family planning services.

## Background

Injectable contraceptives have gained popularity amongst contraceptive users in preventing unintended pregnancies. The proportion of women of reproductive age who have the need for family planning satisfied by modern contraceptive methods including injectable contraceptives (Sustainable Development Goals (SDG) indicator 3.7.1) has increased gradually in recent decades, rising from 73.6% in 2000 to 76.8% in 2020 [1, 2]. In just a decade, its use increased by two folds and it is said to provide protection for more than 42 million women worldwide on a yearly basis [3]. However, more than 200 million women in developing countries wishing to avoid pregnancy do not have access to modern contraceptives [4–7]. Injectable contraceptives like depot-medroxyprogesterone acetate (DMPA) stand as the fifth most commonly used contraceptive method worldwide and the most popular contraceptive method in SSA. It is administered either intramuscularly (IM) or subcutaneously (SC) every 12 weeks or 3 months for pregnancy prevention [8]. Studies revealed that DMPA is safe and effective, and is the preferred choice of more than one in two users of modern contraceptives and one out of three for any contraceptives in SSA [9–11].

Despite being the most widely used method of contraception in SSA, injectable contraceptives are almost provided entirely in clinical settings by trained healthcare staff in developing countries, in contrast to other contraceptives like oral contraceptives pills and condoms which have embraced other delivery options [2, 10]. SSA is known to have the lowest ratio of healthcare staff per population. It is estimated that the global shortage of healthcare staff in SSA will expand from 12 to 18 million by 2030, with a 6 million shortage in Africa [12]. As a result, urban areas enjoy a greater package of trained healthcare personnel as opposed to rural areas [4, 5, 13–15]. Consequently, stark differences exist between rural and urban areas in terms of contraceptive prevalence rate (CPR) and total fertility rate (TFR) [5]. Take for instance, in Zambia, the CPR amongst married women is 42% for a corresponding TFR of 4.3 in urban areas as opposed to a CPR of 28% and a TFR as high as 7.5 in rural areas [16]. In Nigeria, contraceptive prevalence is just 9% in rural areas versus 27% in urban areas [5]. This urban–rural discrepancy would only keep rising if access to family planning (FP) methods, including injectable contraceptives will continue to be restricted to women in well-resourced areas if policies limiting the delivery of injectable contraceptives to clinical settings remain untouchable [6].

It is true that delivery in clinical settings is ideal if and only if health care services are accessible to all women in sub-Saharan Africa irrespective of their location, religion, economic status and educational level. Unfortunately, this scenario remains a far-fetched dream in this context. Will SSA ever meet its needs for family planning, especially with injectable contraceptives being the most preferred method? In the quest to reduce and meet the needs of FP, some countries in SSA have been investing substantially in order to improve access to FP services as well as expanding the contraceptive method mix [4, 10, 14, 15, 17–25]. These two strategies have proven to increase not only contraceptive uptake, but also the overall CPR in resource-poor settings [18]. The governments are increasingly seeking alternative means of delivering injectable contraceptives to rural, hard-to-reach and marginalized population groups in order to achieve the desired goals.

Community health workers (CHWs) have been considered an effective service delivery option, especially in rural areas. Their services involved promoting FP through health education, distributing oral contraceptive pills and condoms, and referring clients for clinic-based services [18, 26]. However, their full potential in expanding method choice has been limited, as the delivery of injectable contraceptives by CHW still remains a hot policy debate in most SSA countries [18]. This, therefore, puts a serious constraint on meeting the needs and preferences of women desiring FP. As a result, there is a pressing need for health policymakers to reconsider injectable contraceptive delivery options as it increasingly overshadows other modern contraceptive methods as the preferred choice by women in SSA.

In 2009, the WHO and United States Agency for International Development (USAID) confirmed that CHW can screen clients effectively, administer DMPA injections safely and counsel on side effects competently as do facility-based providers of progestin-only injectables provided they receive appropriate and competency-based training [27, 28]. Appreciating the available evidence by synthesizing and evaluating primary data on the impact of CHWs in the delivery of injectable contraceptives in SSA countries remains paramount. Malarcher et al. [3] did a systematic review synthesizing evidence on the safety, effectiveness and feasibility of CHWs to administer injectable contraceptives and concluded that, CHWs, if appropriately trained and supervised can safely provide DMPA. However, this study captured only one published study from Africa [19]. Till date, only a few African countries have put in place policies in favour of CHW provision of injectable contraceptives. Madagascar alone has a national policy, allowing injectable contraceptives to be provided by CHWs while other countries like Nigeria, Ethiopia, Senegal, Uganda and Kenya provided special waivers to carry out pilot projects to determine its feasibility [18]. This systematic review, therefore, seeks to: (i) assess the ability of CHWs to provide injectable contraceptives to clients safely; (ii) assess the acceptability of community-based delivery of injectable contraceptives by clients; and (iii) evaluate the effectiveness of the community-based provision of injectable contraceptives.

## Methods

### Study protocol

A protocol which is in line with the Preferred Reporting Items for Systematic Review and Meta-Analysis Protocols (PRISMA) guidelines [29] was developed to guide this systematic review process.

### Search strategy

A comprehensive search strategy was developed and used to identify both published and unpublished studies that were included in this review. This involved a comprehensive search of international electronic databases, websites of organizations, bibliographic searching of included papers and contacting experts in the domain of community-based distribution of injectable contraceptives.

The international electronic databases searched for articles were: the Cochrane Library (CDSR, Wiley), MEDLINE (Ovid), POPLINE, PubMed, CINAHL (Cumulative Index to Nursing and Allied Health Literature), EMBASE (Ovid), Global Health CABI (Ovid), WHO Global Health Library (Global Index Medicus) and Web of Science (Ovid) databases, from inception to 25th March 2022. Articles with Medical subject headings (MESH) and common terms related to community health workers and injectable contraceptives were targeted. Search terms for previous reviews on community health workers and DMPA were adapted as appropriate [28, 30, 31]. All databases were searched thrice for articles, on the 20^th^ May 2017, 29th August 2019 and 25th March 2022. For injectable contraceptives, the search was limited to DMPA and its common terms for two main reasons. Firstly, it is one of the two injectable contraceptives recommended by WHO alongside norethisterone enanthate (NET-EN) for community-based distribution programmes [32]. Secondly, the distribution of NET-EN has been associated with operational difficulties in community settings [3]. No study filter was used during the search process. All articles were stored in the Mendeley library to ease electronic de-duplication.

Websites of organizations known to support community-based reproductive health initiatives in SSA such as CARE International, Population Council, UNFPA, Family Health International (now known as FHI 360) and PATH were also searched for articles [28].

The reference lists of all identified eligible studies were manually checked for studies not identified by the search strategy.

Experts in the domain of community-based provision of contraceptives were contacted for the identification of unpublished literature and any ongoing studies. These experts were contacted/identified through existing published articles since most of those who had done work on the provision of contraceptives to communities in SSA were still working on related projects.

### Language

All studies irrespective of the language of presentation were included in the review.

### Data management and selection process

Records of studies retrieved from all databases were exported to the Mendeley library. In the Mendeley library, they were de-duplicated, screened and managed. The titles and abstracts of these records were screened to assess the full-text copies of potentially eligible studies. Explanations were provided if any potentially eligible study was excluded and the screening was done independently. A total of 4 reviewers were involved in the process of performing title, abstract screening, and full-text reviews and the reviewers declared no conflict of interest.

### Study eligibility criteria

#### Study selection and inclusion

Studies that have evaluated the performance of CHWs in the delivery of injectable contraceptives irrespective of their study designs were included.

#### Participants

Studies that investigated the experience of women of childbearing age (15–49 years) to whom injectable contraceptives have been administered irrespective of their marital status were included.

#### Interventions

Projects or interventions with documented experience of the use of CHWs to deliver injectable contraceptives were included. Community-based strategies in delivering injectable contraceptives such as drug vendors including pharmacies and mobile clinics were excluded.

#### Comparator

Both comparative and non-comparative studies on the provision of injectable contraceptives by CHWs to the provision in clinical settings by trained healthcare staff were considered.

#### Outcomes

Studies that examined safety by assessing the occurrence of injection site morbidities such as numbness, abscesses, severe pain or any other adverse effects reported by participants were included. Adverse effects that required further medical treatment or hospitalization were considered severe. To assess client acceptability, client satisfaction and continuation rates were taken into consideration. Also, studies that included reports of client satisfaction with the intervention that is, the provider (CHW or a trained healthcare staff) and the overall satisfaction of the contraceptive method were included as well as those that included the continuation rates of women with the method and provider. Finally, studies that reported the uptake of DMPA services during the intervention in terms of new users to family planning, new users of DMPA, returning users of DMPA and clients switching from clinic-based provision to community-based provision or vice versa were equally included.

#### Timing

A follow-up period of at least 3 months was a pre-requisite for a study to be eligible for inclusion.

#### Exclusion criterion

One review was excluded at the full-text phase and all studies that did not meet the inclusion criteria were excluded in this study.

### Data extraction

A customized data collection form was developed and tested on two included studies. The form was adapted and used to extract relevant study data from full-text papers of included studies. The data extraction form was completed electronically in order to minimize transcription error.

### Data items

Data were summarized using tables. The data included information such as the author’s name, year of publication and country of origin, the methodology used (study design and study period), the participants (setting, sample size and demographic characteristics of DMPA providers and recipients), the intervention, the study outcomes and the risk of bias assessment. Also, data extraction was done independently.

### Quality assessment

The Effective Public Health Practice Project (EPHPP) tool [33] was used to assess the quality and the risk of bias of studies included. This tool is appropriate for assessing all study designs and it has been independently evaluated and judged suitable for use in appraising articles for systematic reviews [34, 35]. All studies were assessed individually for quality with no customizations made to the EPHPP tool. Guidance and clarification were sought from the EPHPP quality assessment dictionary [36]. Quality assessment was done independently without arbitration.

### Data synthesis

The variety of the study designs and poor study qualities made meta-analysis an inappropriate choice for data synthesis. As a result, a narrative synthesis was undertaken with guidance from the proposed guideline by the Economic and Social Research Council (ESRC) methods programme [37]. The study characteristics, participants’ characteristics and study results of included studies are described and summarized in tables.

## Results

### Study selection

A total of 1358 studies were identified by the search strategy, 1341 from 9 electronic databases and 17 from grey literature sources. All studies from grey literature sources were retrieved from the websites of organizations that have been involved in promoting community-based reproductive health programmes in SSA.

**Fig. 1:**
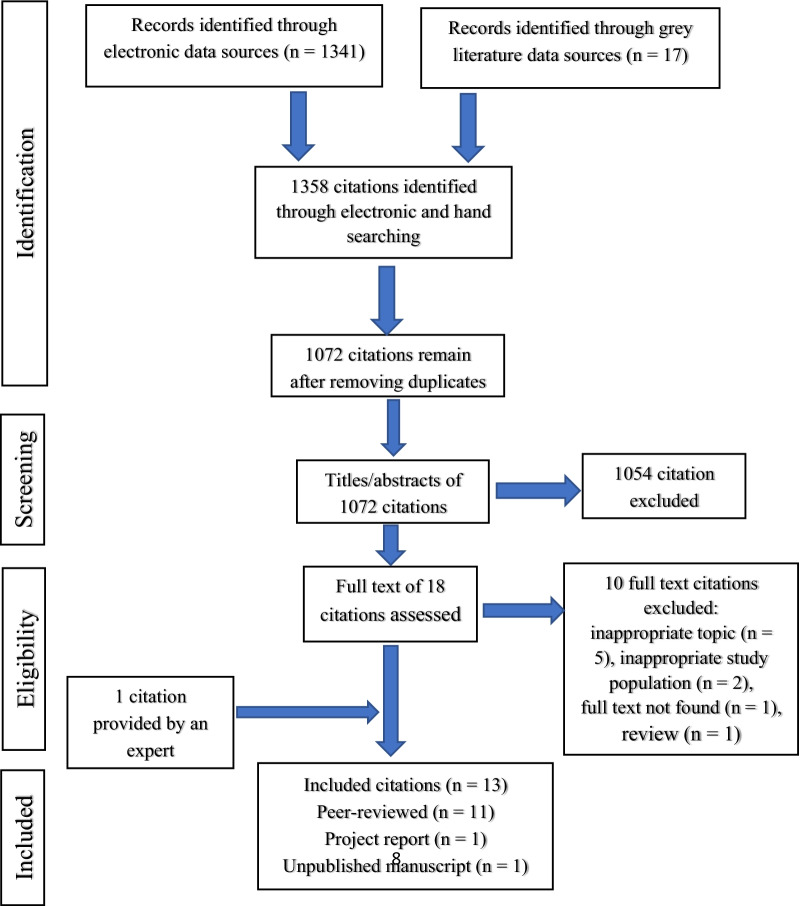
Flowchart of the study selection process

A manual enhancement of the de-duplication with the Mendeley software led to the identification and removal of 86 duplicate sets containing 286 citations. A further 1054 citations were removed after screening the titles and abstracts of the remaining studies and a full-text review of 20 studies were reviewed. Of the 20 studies, 12 met the inclusion criteria and the identification of 1 unpublished study also met the inclusion criteria [20] giving a total of 13 studies included in this review, all published in English. The detailed flowchart of the study selection process is shown in Fig. 1.

### Study characteristics

#### Study types and designs

A wide variety of study designs have been used to examine CHW performances in SSA based on the present evidence base. Of the 13 eligible studies, 4 were non-randomized community trials (non-RCTs) [19–22], 3 descriptive cross-sectional studies [4, 15, 18], 2 descriptive longitudinal studies [23, 24], 1 pilot intervention [14], 1 randomized control trial [17] and 2 case study [10, 25]. Most of the studies had a study period of 12–13 months [15, 21, 23, 25] and 1 had a study period of 3 years [20].

#### Year of publication

All articles reviewed were published between 2007 and 2022. Thus, indicating that the potential contributions of CHWs to the delivery of DMPA in SSA are still in their early stages.

#### Sample size

The sample size varied greatly across studies, ranging from 252 to 35,000. A priori estimation of sample sizes was observed in the 4 non-RCTs. Three studies interviewed a handful of participants who were randomly selected from all the recruited DMPA clients [15, 18, 24].

Attrition rates were not reported for any of the longitudinal study designs. However, all the non-RCTs mentioned the occurrence of client drop-outs. Of these, only two reported attrition rates of 13.8% [22] and 17.8% [19].

#### Settings and country of origin

These studies were carried out in rural areas/regions. More specifically in regions classified by the African Union or United Nations SSA regions (Western, Eastern, and Central Africa) [38]. The Western African region included 1 study in Senegal [24] and 1 in the Republic of Benin [4]. The Eastern African region included 3 studies in Uganda [19, 20, 25], 2 in Ethiopia [10, 21], 1 in Kenya [23], 1 in Zambia [15], 1 in Mozambique [22] and 2 in Madagascar [17, 18]. The Central African region included 1 study in the Democratic Republic of the Congo [14].

#### DMPA service providers

The CHWs were identified with different names across studies. Seven studies identified them as CHWs [4, 10, 14, 15, 17, 23, 25], two as community-based reproductive health workers [19, 20], one as matrons [24] and one as community-based reproductive health agents [24]. These characters were all referred to as CHWs in this review.

All community-based DMPA service providers were chosen from a pool of CHWs. Most CHWs were women in the selected studies [18–20, 22, 24]. Agentes Polyvalent Elementary (APEs) in Mozambique, on the other hand, was dominated by men (17 men: 8 women) [22] and 8 studies failed to include information on gender distribution [4, 5, 10, 14, 17, 21, 23, 25].

CHWs were compensated in a variety of ways. The CHWs in Madagascar were given a modest sum from each DMPA sold [18], APEs in Mozambique were paid monthly by the government and traditional birth attendants (TBA) received in-kind contributions from clients [22] while in Uganda, non-governmental organizations (NGOs) gave periodic gifts [19, 20].

The ages and years of experience of CHWs were not reported in most studies. However, the age range of CHWs was 20–55 years with the range of years of experience being 5–120 months in Madagascar [18]. In Senegal, the mean age of CHWs was 40 years and the mean years of experience were 12 years [24].

#### DMPA recipients

Two descriptive studies reported on the age range of DMPA recipients, while 1 study reported on the mean age. The age range provided ranged from 15–53 years and the average age was 31 years [14, 15, 18, 24]. The mean age of clinic-based clients in non-RCTs varied between 26 and 28 years [19–21], while that of CHWs clients varied between 28 and 30 years [19–21].

The range of the number of living children for DMPA clients was reported in two descriptive studies. In these studies, the number of living children ranges from 0–14 [15, 18]. Chin-Quee et al. [15]; Family Health International-United States Agency for International Development (FHI-USAID) [24] reported the mean number of children to be 3.6 and 4.5, respectively. The non-RCTs of Stanback et al. [19]; Prata et al. [21]; Jacinto et al. [22] reported the mean number of children for comparative groups to be 4–8 children for CHWs clients and 3.6 to 3.9 for clinic-based clients.

Amongst the descriptive studies, Mwembo et al. [14] and FHI-USAID [24] reported the marital status of DMPA clients with 78.6% and 96% of clients being married. For non-RCTs, the proportion of married CHW clients varied from 83% in Mozambique [22] to 88% in Ethiopia [21]. For clinic-based clients, it varied from 80% in Uganda [19] to 92.4% in Ethiopia [21].

Studies on non-RCTs reported the educational levels of clients. In these studies, more clinic-based clients had not received formal education compared to CHW clients, 92.4% versus 89.4% [21] and 16% versus 8% [19, 20]. In the study, conducted by Jacinto et al. [22], 60% of the clients served by CHWs had no formal education while in the study by Mwembo et al. [14], 73.3% had formal education.

More than 75% of clients declared that their husbands are in support of them taking DMPA in Mozambique [22] while in Uganda, 41% of clients served by CHWs had the support of their husbands compared to 52% of clinic-based clients [19].

#### Interventions

The 11 studies involved the administration of DMPA intramuscularly and 2 subcutaneously [4, 14]. The implementation of each study was carried out by both government workers and non-governmental organizations. Amongst the 13 studies, 12 were pilot studies and 1 was a scaled-up initiative [25].

Prior to implementation, CHWs and their supervisors underwent in-service training. The duration of the training varied from 3 days in Madagascar to 4 weeks in Zambia, consisting of a classroom and practicum component in clinics. The training content was similar across all studies, for example in Ethiopia, it consisted of family planning (FP) methods, study protocol, screening, injection administration, infection prevention, safe injection technique and reporting procedures. Olawo et al. [23] reported supplying job aids to CHWs to facilitate the determination of client’s eligibility and the detection of contraindications for DMPA administration in Kenya. No study made mention of refresher training sessions for CHWs. The number of CHWs trained ranged from 20 in Uganda [19, 20] to 61 in Madagascar [18].

In most studies, DMPA was administered in CHWs' homes or clients’ homes or in determined places in the community [15, 19–22]. In Senegal, matrons served clients in health huts [24]. The service delivery point was not clear in the study done by Krueger et al. [25]. DMPA was offered to clients for free in the study conducted by Hoke et al. [18]. The clinic-based clients received DMPA in clinics for the studies that were clinic-based.

Close supervision of CHWs in the 13 studies was ensured by trained facility-based providers (nurses and physicians). The supervisors were responsible for monitoring competency, ensuring service records were complete and properly filled and reinforcing DMPA provision skills.

#### Outcomes and outcome measures

Safety, acceptability and effectiveness (the ability of CHWs to well provide DMPA) of CHW's provision of DMPA were assessed using the following outcomes: injection site morbidity for safety, client satisfaction and continuation rates for acceptability and the uptake of DMPA services for effectiveness. The uptake of DMPA services was further sub-divided into 4 other outcomes; new users of family planning, new users of DMPA, returning users of DMPA and clients of DMPA who desire to switch providers or those who switched providers in the course of the study.

#### Descriptive studies

The data for studies that examine safety, continuation rates, and client satisfaction came from CHWs reports [23, 24, 34], client registers [25], and client interviews [15]. FHI-USAID [24] did not report on safety. The data for uptake of DMPA services were obtained from client interviews and family planning service statistics [15], CHW reports [18, 23, 24], client registers and family planning service statistics [25]. Family planning service statistics were obtained from health districts to which CHWs were affiliated.

Four studies collected data once, but at different time points during or after the implementation phase. Data were collected in the 9^th^ month in Zambia [15], the 7^th^ month in Madagascar (at the end of the implementation phase) [18] and during the 1-year period of implementation [15]. In Kenya, the data were collected at the 3rd, 6th and 9th months [26], while in Senegal the data were collected at the 3rd and 7th months [24].

#### Non-randomized community trials (non-RCTs)

Data for all outcomes were collected through client surveys with a predesigned questionnaire. As per Jacinto et al. [22]; Prata et al. [21] studies, the data were collected at the enrolment, 13th week and 6 months while the data were collected at the enrolment and 13th week in the study by Stanback et al. [19] and at 3 years in the study by Poss et al. [20].

#### Quality assessment and risk of bias in studies

Each study was evaluated across 6 categories namely; selection bias, study design, confounders, blinding, data collection methods and withdrawals or drop-outs and given a score of 1 to 3 accordingly. The evaluation of withdrawals or drop-outs was not appropriate for cross-sectional studies [15, 18] and the case study [10, 25], hence was not assessed for these studies. Each category was assigned an overall rating of weak, moderate or strong for 1, 2 or 3, respectively. Only 2 studies controlled for confounding during data analysis [19, 20].

Most of the studies were rated to be of weak quality. A summary table of the quality assessment of the reviewed studies is shown in Table 1.

**Table 1:** Quality assessment of included studies

Reference, country	Overall quality assessments	Components of quality assessment					
		Selection bias^a^	Study Design	Confounding^b^	Blinding	Data collection method^c^	Withdrawals and drop-outs
Weidert et al. [10] Ethiopia	Moderate	Strong	Strong	Weak	Weak	Moderate	NA
Okegbe et al. [4] Republic of Benin	Moderate	Strong	Strong	Weak	Weak	Moderate	NA
Mwembo et al. [14] Democratic Republic of Congo	Moderate	Moderate	Strong	Weak	Weak	Moderate	Moderate
Comfort et al. [17] Madagascar	Weak	Weak	Moderate	Weak	Weak	Moderate	NA
Prata et al. [21] Ethiopia	Weak	Weak	Strong	Weak	Weak	Moderate	Moderate
Stanback et al. [19] Uganda	Weak	Weak	Strong	Strong	Weak	Moderate	Strong
Krueger et al. [25] Uganda	Weak	Weak	Moderate	Weak	Weak	Strong	NA
Hoke et al. [18] Madagascar	Weak	Strong	Moderate	Weak	Weak	Moderate	NA
Jacinto et al. [22] Mozambique	Weak	Strong	Strong	Weak	Weak	Moderate	Strong
Chin-quee et al. [15] Zambia	Weak	Strong	Moderate	Weak	Weak	Moderate	NA
Olawo et al. [23] Kenya	Weak	Weak	Moderate	Weak	Weak	Strong	Strong
FHI-USAID [24] Senegal	Weak	Strong	Moderate	Weak	Weak	Strong	Strong
Poss et al. [20] Uganda	Weak	Weak	Moderate	Strong	Weak	Moderate	Weak

^a^For observational studies, likelihood of bias was rated based on how likely the participants can be representative of the target population or for non-RCT rated as ‘very likely’ to be representative of target population if not referred from a source and not self-referred

^b^Assessment of confounders was based on how far authors went to control confounding by design or in their analyses

^c^Data collection method rated as strong if from medical records and moderate or weak if from self-reported data or assessment/screening by researchers Guidance taken from EPHPP Quality Assessment Tool for Quantitative Studies Dictionary

#### Safety

Injection site morbidity as reported by supervisors and DMPA clients was satisfactorily low in most of the studies. The highest morbidity rates were observed in the Democratic Republic of Congo 40.6% [14] and Madagascar 3% [18]. In Uganda, 0.7% and 0.3% of injection site morbidity was reported [19, 25]. However, a significant difference was observed in Ethiopia as more clients of CHWs reported induration at injection sites than clinic-based clients at the 13th week of data collection, 2.1% versus 0.5%, respectively [21]. Zambia [15], reported about 2% of injection site morbidity while Mozambique [22] reported less than 0.5%.

#### Uptake of DMPA services

CHW's provision of injectable contraceptives to communities exposed many women to FP services. Six studies reported the proportion of women who were receiving a FP method for the very first time [4, 15, 18, 22–24]. The proportion of new FP users served by CHWs ranged from 14% in Kenya [26] to 66% (APE's clients) in Mozambique [25] and 97.8% in the Republic of Benin [4].

Two studies that compared CHW services to clinic-based services showed significant differences between these two for reaching out to new DMPA clients, 86% versus 76%, respectively, in Uganda [19] and 58.4% versus 45.9%, respectively, in Ethiopia [21]. However, one study showed higher proportions of DMPA users in clinics compared to CHWs, 57% versus 50%, respectively [25].

During the implementation phase of some programmes, women who initially received DMPA from clinics switched CHWs as providers or express their desire to have CHWs as providers. In the Republic of Benin, 54% of women switched from clinic providers to CHWs [4] while in Senegal, 21% of women switched from clinics to CHWs [24] and in Zambia, 20% of women expressed their wish to be served DMPA by CHWs [15] and in Kenya, 74% of women opted to switch to CHWs as providers [23].

In comparison studies, 94.6% of women in the Democratic Republic of Congo expressed interest to continue receiving DMPA in the community by a CHW rather than in a health facility [14]. About 52% of clinic clients in Ethiopia expressed their interest to be served by CHWs [21] and in Uganda, 38.5% of CHWs clients switched to clinics for DMPA provision as opposed to 41.5% of clinic clients who switched to CHWs for DMPA [20].

Some programmes also created the opportunity for past users of DMPA to recommence DMPA as a contraceptive method [18, 21, 22]. In Ethiopia, 34% of CHWs clients were returning users as opposed to 44% of clinic clients [21]. The summary of findings on the effectiveness and safety of CHW provision of DMPA in several countries of SSA and in some instances, in clinical settings, is provided in Tables 2 and 3, respectively.

**Table 2:** Summary of findings of the effectiveness of CHW provision of DMPA in several countries of SSA and in some instances, in clinical settings

DMPA uptake							
Author, country	Mode of outcome measurement	Provider	Number of clients	New to family planning	New to DMPA	Returning users	Desire to switch provider
Weidert et al. [10] Ethiopia	CHW reports	CHW	8604	19%	25%	NR	NR
Okegbe et al. [4] Republic of Benin	CHW reports	CHW	35,000	80%	22.8%	NR	54%
Mwembo et al. [14] Democratic Republic of Congo	CHW reports	CHW	252	70%	69.8%	NR	NR
Comfort et al. [17] Madagascar	CHW reports	CHW	622	NA	NA	NA	NA
Chin-quee et al. [15] Zambia	Client interview	CHW	3479	42.5%	24%	NR	20%
Hoke et al. [18] Madagascar	CHW reports	CHW	303	28%	25%	50%	NR
Olawo et al. [23] Kenya	CHW reports	CHW	832	14%	12%	NR	74%
FHI-USAID [24] Senegal	CHW reports	CHW	308	64%	15%	NR	21%***
Krueger et al. [25] Uganda	Client registers	CHW CLINIC	1364 457	NR NR	30% 57%	NR NR	NR NR
Prata et al. [21] Ethiopia	Client surveys at enrolment, 13^th^ week and 6 months	CHW CLINIC	622 440	NR NR	58.4%** 45.9%	34% 44%	0 52%
Stanback et al. [19] Uganda	Client surveys at enrolment, 13^th^ week	CHW CLINIC	449 328	NR NR	86% 76%	NR NR	NR NR
Poss et al. [20] Uganda	Client surveys at 3^rd^ year	CHW CLINIC	308 217	NR NR	NR NR	NR NR	38.9% 41.5%
Jacinto et al. [22] Mozambique	Client surveys at enrolment, 13th week and 6 months	CHW (TBA) CHW (APE)	782 649	63% 66%	NR NR	30%	NR NR

*CHW* community health workers, *TBA* traditional birth attendants, *APE* Agentes polyvalent elementaries, *NA* not applicable, *NR *not reported

*Satisfaction rates at 3 months and 6 months, respectively, **p < 0.05, ***switched to CHW

**Table 3:** Summary of findings of the safety of CHW provision of DMPA in several countries of SSA and in some instances, in clinical settings

Safety			
Author, country	Mode of outcome measurement	Provider	Injection site morbidity
Weidert et al. [10] Ethiopia	CHW reports	CHW	NR
Okegbe et al. [4] Republic of Benin	CHW reports	CHW	NR
Mwembo et al. [14] Democratic Republic of Congo	CHW reports	CHW	40.6%
Comfort et al. [17] Madagascar	CHW reports	CHW	NA
Chin-quee et al. [15] Zambia	Client interview	CHW	2%
Hoke et al. [18] Madagascar	CHW reports	CHW	3%
Olawo et al. [23] Kenya	CHW reports	CHW	0
FHI-USAID [24] Senegal	CHW reports	CHW	NR
Krueger et al. [25] Uganda	Client registers	CHW CLINIC	0 0
Prata et al. [21] Ethiopia	Client surveys at enrolment, 13th week and 6 months	CHW CLINIC	2.1% 0.5%
Stanback et al. [19] Uganda	Client surveys at enrolment, 13th week	CHW CLINIC	0.7% 0.3%
Poss et al. [20] Uganda	Client surveys at 3rd year	CHW CLINIC	NA NA
Jacinto et al. [22] Mozambique	Client surveys at enrolment, 13th week and 6 months	CHW (TBA) CHW (APE)	< 0.5%

#### Continuation rates

Out of the 13 studies reviewed, 2 reported continuation rates of DMPA from 3 to 9 months [23, 25]. High levels of continuation rates at 3 months were observed for CHWs clients who had received the first injection of DMPA in most studies, ranging from 68.1% in Mozambique [22] to 96% in Madagascar [18] and from 68% in Kenya [23], 84% in Uganda [25] and 94.8% in Democratic Republic of Congo [14].

The study conducted by Stanback et al. [19]; Prata et al. [21] compared the rates amongst clients of clinics and CHWs with one showing significant differences in favour of CHWs at both 3 months (83.7% versus 81.6%) and 6 months (78.8% versus 62.3%) [21]. The summary of findings for continuation rates of CHW provision of DMPA in several countries of SSA and in some instances, in clinical settings, is shown in Table 4.

**Table 4:** Summary of findings for continuation rate of CHW provision of DMPA in several countries of SSA and in some instances, in clinical settings

Continuation rates						
Author, country	Mode of outcome measurement	Provider	Number of women, 1st injection	3 months	6 months	9 months
Weidert et al. [10] Ethiopia	CHW reports	CHW	8604	NR	NR	NR
Okegbe et al. [4] Republic of Benin	CHW reports	CHW	7997	NR	NR	NR
Mwembo et al. [14] Democratic Republic of Congo	CHW reports	CHW	252	94.8%	92.1%	NR
Comfort et al. [17] Madagascar	CHW reports	CHW	NR	NA	NA	NA
Chin-quee et al. [15] Zambia	Client interview	CHW	253	94%	NR	NR
Hoke et al. [18] Madagascar	CHW reports	CHW	303	96%	NR	NA
Olawo et al. [23] Kenya	CHW reports	CHW	832	89%	81%	68%
FHI-USAID [24] Senegal	CHW reports	CHW	308	93%	NR	NR
Krueger et al. [25] Uganda	Client registers	CHW CLINIC	NR NR	72% NA	70% 57%	84% NA
Prata et al. [21] Ethiopia	Client surveys at enrolment, 13^th^ week and 6 months	CHW CLINIC	622 440	83.7%** 81.6%	78.8%** 62.3%	NR NR
Stanback et al. [19] Uganda	Client surveys at enrolment, 13th week	CHW CLINIC	449 328	88% 85%	NR NR	NR NR
Jacinto et al. [22] Mozambique	Client surveys at enrolment, 13th week and 6 months	CHW (TBA) CHW (APE)	782 649	80% 68.1%	91.6% 68.6%	NR NR

*CHW *community health workers, *TBA* traditional birth attendants, *APE* Agentes polyvalent elementaries, *NA *not applicable, *NR n*ot reported

*Satisfaction rates at 3 months and 6 months, respectively, **p < 0.05, ***switched to CHW

#### Client satisfaction

Client satisfaction with CHW as a provider and DMPA as a contraceptive method was high (at least 70%) in most cases that reported this outcome [10, 14, 15, 18, 19, 21, 22]. Non-RCT studies in Uganda and Ethiopia did not report any differences between the satisfaction rates of CHW clients and clinic-based clients [19, 21]. However, a significant difference was observed in client satisfaction rates between the two types of CHWs [22]. At the 2nd round of data collection (3 months), clients of APEs expressed significant satisfactory rates compared to TBAs clients (89.1% versus 73.7%). Nevertheless, the differences in satisfactory rates were greatly reduced by the 3rd round (6 months) of data collection (94.1% versus 89.8%).

Okegbe et al. [4]; Comfort et al. [17]; Fhi-Usaid [24] did not examine client satisfaction for DMPA as a contraceptive method and Olawo et al. [23] did not report client satisfaction. The summary of findings for satisfaction rates of CHW provision of DMPA in several countries of SSA and in some instances, in clinical settings is shown in Table 5.

**Table 5:** Summary of findings for satisfaction rates of CHW provision of DMPA in several countries of SSA and in some instances, in clinical settings

Satisfaction					
Author, country	Mode of outcome measurement	Provider	Number of clients	With DMPA	With provider
Weidert et al. [10] Ethiopia	CHW reports	CHW	8604	47.8%	39.4%
okegbe et al. [4] Republic of Benin	CHW reports	CHW	35,000	22.8%	NR
Mwembo et al. [14] Democratic Republic of Congo	CHW reports	CHW	252	96.1%	87%
Comfort et al. [17] Madagascar	CHW reports	CHW	622	NA	NA
Chin-Quee et al. [15] Zambia	Client interview	CHW	253	94%	NR
Hoke et al. [18] Madagascar	CHW reports	CHW	303	96%	NR
Olawo et al. [23] Kenya	CHW reports	CHW	832	NR	81%
Fhi-Usaid [24] Senegal	CHW reports	CHW	308	NR	NR
Prata et al. [21] Ethiopia	Client surveys at enrolment, 13^th^ week and 6 months	CHW CLINIC	622 440	99.2% (98.8%)* 98.4% (100%)*	95.6% (96.2%)* 97.6% (97.6%)*
Stanback et al. [19] Uganda	Client surveys at enrolment, 13^th^ week	CHW CLINIC	449 328	93% 90%	95% 93%
Jacinto et al. [22] Mozambique	Client surveys at enrolment, 13^th^ week and 6 months	CHW (TBA) CHW (APE)	782 649	74.7% (90.1%)* 88.2% (89.2%)*	73.7% (89.8%)* 89.1% (94.1%)*

*CHW *community health workers, *TBA* traditional birth attendants, *APE* Agentes polyvalent elementaries, *NA* not applicable, *NR* not reported

*Satisfaction rates at 3 months and 6 months, respectively, **p < 0.05, ***switched to CHW

## Discussion

This systematic review included 12 empirical research articles and 1 unpublished manuscript which resulted from the application of the inclusion criteria to the findings of a comprehensive search strategy, a surprisingly small number given the high unmet family needs and the popularity of injectable contraceptives in SSA. Nevertheless, the supplementation of the electronic database search results with hand searching, searching of the reference lists of included articles and contacting experts (authors of included studies) provides the confidence that all the existing relevant studies were captured. The findings generated by this systematic review are based on the synthesis of all available evidence.

The study revealed that there were significant differences amongst clients who were using DMPA for the first time [4, 10, 14, 19, 21], clients wanting to switch providers [21] and those continuing with DMPA after the second injection (3^rd^ month) and third injections (6th month) [21], all in favour of CHWs. A study demonstrated a significant difference in the reports of injection site morbidities by clients after receiving the second injection of DMPA in favour of trained health care personnel [21]. However, injection site morbidities were uneventful after the 3rd injection.

This review provides evidence that the use of trained CHWs to deliver injectable contraceptives such as DMPA is accepted in SSA communities. Satisfactory results of safety and the uptake of DMPA services by the studied communities were observed across all studies.

Even though all the included studies portray satisfactory performance levels of trained CHWs in delivering DMPA within communities, the evaluation of the overall performance of CHWs should be taken with a lot of caution as the included studies were generally of poor quality.

### Interpretation of findings in the light of other literature

Malarcher et al. [3] conducted a review on the evidence of the performance of CHWs in the provision of injectable contraceptives around the world. The possibility of conducting a meta-analysis, as with this review, was not feasible due to insufficient information in the study designs and methodology (resulting in low internal validity ratings). Malarcher et al. [3] obtained similar results to this review, demonstrating that CHWs were competent in delivering injectable contraceptives to the community. According to Malarcher et al. [3]; Okegbe et al. [4]; Weidert et al. [10]; Mwembo et al. [14], CHWs can screen clients and provide DMPA safely with similar levels of competency as clinic-based staff under appropriate training and supervision. Also, client satisfaction with CHW services was high, and there was an overall increase in the use of FP services.

However, the evidence gathered from SSA included in this review represented a small number of studies. Despite respecting the different stages of conducting a systematic review, the review by Malarcher et al. [3] had some flaws. The inclusion of evidence from personal communication with project staff was highly prone to recall bias. Besides, they did not precise the role played by these project staff in the course of the project and whether they were interviewed during the course of the project or not. Furthermore, the weight of the evidence provided by the preliminary project report of Prata et al. [21] will not be the same as that of a completed project report.

Malarcher et al. [3] presented one-year continuation rates of 55% and 44% for CHWs clients and clinic clients, respectively. Unfortunately, these figures were not seen in the referenced studies, that is the studies by Stanback et al. [19] and Poss et al. [20].

In the light of this appraisal, it is clear there is a need for a more rigorous and clearly reported systematic review to assess the current evidence base on the potential of CHWs to deliver injectable contraceptives. However, the focus of this review was limited to SSA as it is the region with the highest unmet needs of FP and is also faced with appalling shortages in the health workforce [39]. In addition, conducting this current review in 2017 permitted the inclusion of 5 more papers recently published [15, 22–25] and the completed project report by Prata et al. [21] in Ethiopia.

### A possible explanation of the findings

In SSA, the provision for CHWs appears to be a safe, acceptable, and effective alternative to health provider delivery of injectable contraceptives. Nonetheless, several considerations should be made for implementation and future research, particularly given the scarcity of research in SSA.

The use of syringes outside of health facilities and community-based injectable contraceptive provision by CHWs has raised concerns. Also, concerns have been raised about the consequences of improper needle handling and disposal, such as the spread of blood-borne diseases such as hepatitis and HIV/AIDS [6]. However, according to WHO technical guidelines, trained CHWs can safely administer intramuscular and subcutaneous injections [27, 40].

In this review, the safety of the administration of injectable contraceptives by CHWs was assessed by the occurrence of injection site morbidity. The prevalence of injection site morbidity was generally low and similar for both types of providers. The study by Prata et al. [21] showed significant differences between the two types of providers, after the administration of the 2nd injection (3 months). However, the occurrence of injection site morbidity was non-existent by the next follow-up survey, i.e. after administration of the 3rd injection (6 months). This implies that through continuous practice, CHWs improved their mastery of safe injection techniques. Moreover, their skills may have been reinforced during project implementation through formative supervision and refresher training sessions. Take for instance, in Kenya, CHWs spent an entire day once a week administering DMPA to clients in health facilities under supervision [23]. It is therefore not surprising that these CHWs did not register any injection site morbidities.

DMPA clients were generally very satisfied with both CHWs and clinic-based workers as providers of DMPA as well as DMPA for a contraceptive method. Three studies reported very high satisfaction rates for CHWs as providers, 100% was observed in Ethiopia [21], 98% in Kenya [15] and 99% in Senegal [24]. The type of incentives given to CHWs might have played a very important role in motivating CHWs to dispense quality services and also indirectly determined client satisfaction rates. For example, in Ethiopia where client satisfaction was rated at 100%, CHWs received a modest sum for each DMPA sold and administered to clients. This is in line with research carried out by Ramirez [41] which demonstrated that CHWs who received financial incentives experienced lower drop-out rates and dispensed better-quality services compared to those who were unpaid. On the other hand, other studies used different methods of incentivizing, such as gifts, or CHWs who were already involved in community-based family planning programmes and still found high satisfaction rates. Hence, providing some kind of incentives to CHWs directly influences the quality of services provided to clients, resulting in better client satisfaction rates.

Also, DMPA clients were very satisfied with DMPA as a contraceptive method, most likely because none of these women had an unwanted pregnancy (method failure). However, none of the included studies that reported this outcome followed women for more than 9 months, thus, we cannot be sure if some women were able to avoid pregnancy after the study period. Moreover, this outcome was not reported in the study of Poss et al. [20], which was a 3-year follow-up to the Stanback et al. [19] study.

Continuation rates of CHWs clients were high across all studies. Disparities in continuation rates were seen between the two groups of providers in Ethiopia, where a greater proportion of CHW's clients continued with DMPA throughout the study period [21]. These differences can be explained by the fact that clinic-based clients are more likely to forget their subsequent DMPA injection appointment dates, whereas CHWs clients generally receive their injections in their homes or areas close to their homes, with CHWs serving as constant reminders to these women. The means of getting to clinics may also be a contributing factor. Studies have shown that the prevalence of contraceptive use decreases with distance from service delivery points [42]. However, clients self-selected their providers, but one would normally expect clinic-based clients to live close to clinics, or perhaps chose clinic services based on previous experiences with clinic-based providers. Surprisingly, one study reported a higher continuation rate at 9 months than the continuation rates at 3 and 6 months [25]. This disparity occurred because some of the clinic-based clients switched to CHWs as providers (21%) and were therefore considered in the bulk of eligible clients (CHWs clients) for the 4th injection (9 months). Also, Jacinto et al. [22] reported a higher continuation rate at 6 months than at 3 months for both types of CHWs. The clients who were considered lost to follow-up because they were not interviewed during the 13th week after receiving the second DMPA injection, were captured later on during the 6 months survey following the administration of the 3rd injection.

The continuation rates have also been affected by gender barriers. A significant difference in continuation rates was observed between the two groups of CHWs in Mozambique, with one group made entirely of women (TBA) and the other predominantly men (APE). The study of Jacinto et al. [22] showed higher continuation rates for TBAs than APEs. In Mozambique, TBAs served as advisors in sexual and reproductive health in rural communities than APEs.

This study showed that the provision of DMPA by CHWs improved both access to family planning services and method choice in underserved or difficult-to-reach populations. The uptake of DMPA services provided by CHWs was significant across all studies, indicating that DMPA injections delivered by CHWs are not only acceptable but also very effective. Hence, CHWs are a potential solution for increasing population access to contraceptive services, particularly injectable contraceptives.

## Conclusion

Findings suggest that with appropriate training and supervision, CHWs can safely provide injectable contraceptives with little or no needle stick injuries and with rates equivalent to clinic-based providers. Beneficiaries also expressed satisfaction in their services and registered acceptable continuation rates with CHWs. This review demonstrated that CHWs can provide access to women who had never used any family planning method or DMPA before, thereby improving the overall uptake of family planning.

However, the quality of the current evidence base is low in most studies and in order to determine whether the findings are generalizable and reproducible, additional well-designed studies are required. Future studies should be of good quality design, preferably RCTs, present disaggregated data and should include longer follow-up times. It is important to carry out qualitative studies to understand the gender preferences of clients as well as the community perceptions of family planning, especially in countries with no known community-based family planning programmes.

## Data Availability

The data sets during and/or analysed during the current study are available from the corresponding author on reasonable request.
